# Policy Changes to Implement Intramural Sports in North Carolina Middle Schools: Simulated Effects on Sports Participation Rates and Physical Activity Intensity, 2008–2009

**DOI:** 10.5888/pcd11.130195

**Published:** 2014-01-16

**Authors:** Michael B. Edwards, Michael A. Kanters, Jason N. Bocarro

**Affiliations:** Author Affiliations: Michael A. Kanters, PhD, Jason N. Bocarro, PhD, North Carolina State University, Raleigh, North Carolina.

## Abstract

**Introduction:**

Extracurricular school sports programs can provide adolescents, including those who are economically disadvantaged, with opportunities to engage in physical activity. Although current models favor more exclusionary interscholastic sports, a better understanding is needed of the potential effects of providing alternative school sports options, such as more inclusive intramural sports. The purpose of this study was to simulate the potential effect of implementing intramural sports programs in North Carolina middle schools on both the rates of sports participation and on energy expenditure related to physical activity levels.

**Methods:**

Simulations were conducted by using a school-level data set developed by integrating data from multiple sources. Baseline rates of sports participation were extrapolated from individual-level data that were based on school-level characteristics. A regression model was estimated by using the simulated baseline school-level sample. Participation rates and related energy expenditure for schools were calculated on the basis of 2 policy change scenarios.

**Results:**

Currently, 37.2% of school sports participants are economically disadvantaged. Simulations suggested that policy changes to implement intramural sports along with interscholastic sports could result in more than 43,000 new sports participants statewide, of which 64.5% would be economically disadvantaged students. This estimate represents a 36.75% increase in economically disadvantaged participants. Adding intramural sports to existing interscholastic sports programs at all middle schools in North Carolina could have an annual effect of an additional 819,892.65 kilogram calories expended statewide.

**Conclusion:**

Implementing intramural sports may provide economically disadvantaged students more access to sports, thus reducing disparities in access to school sports while increasing overall physical activity levels among all children.

## Introduction

Physical inactivity among adolescents is a significant public health concern in the United States (1). In addition to reducing overweight and obesity, physical activity decreases the risk of chronic diseases (eg, some cancers, heart disease) and improves mental health, independent of physical activity’s relationship to weight status (2,3). Sports participation is essential for promoting adolescent physical activity (4,5) and is a modest predictor of physical activity and healthy lifestyle in adulthood (6).

For many adolescents, especially those from economically disadvantaged backgrounds, school-based extracurricular sports programs provide the best, if not the only, opportunity for sports participation (7). However, evidence suggests that economically disadvantaged students are less likely than their better-off peers to participate in school sports (8). Although the majority of adolescents want to play more school sports, policies favor highly competitive and exclusionary interscholastic sports that restrict participation to only the best athletes and often require many resources from participants (9,10). For example, school sports teams often require a large time commitment and, increasingly, have fees for participation (ie, pay-for-play policies) (11,12). Because most middle schools in North America offer competitive interscholastic sports exclusively (10), a substantial number of students are left on the sidelines. Additionally, sports programs that focus on skill development and competition (eg, interscholastic sport) may not provide adequate levels of physical activity for adolescents (13,14).

Researchers and policy advocates (eg, Institute of Medicine, National Association for Sport and Physical Activity) have called for policy changes to include intramural sports to increase participation and better promote physical activity in school sports programs (15–17). Intramural sports fundamentally differ from interscholastic sports in that they provide a wider range of activities and are open to all students, including those who may lack the time or athletic ability required to participate in interscholastic programs. Additionally, intramural programs are less expensive to organize than interscholastic programs and may require lower fees from participants. However, before changing any policy, it is beneficial to understand the potential effects of proposed policy changes (18). The purpose of this study was to simulate the effect of implementing intramural sports programs in North Carolina middle schools on both the rates of sports participation and the energy expenditure related to physical activity levels. To estimate the effect of this policy change, we conducted simulations by using data from multiple sources.

## Methods

### Data sources

Two primary data sets, identified henceforth as data set 1 (D1) and data set 2 (D2) were used as source data for simulations. The primary individual-level data and estimations of participation rates were derived from survey data obtained in 4 North Carolina middle schools in an urban school district (D1) (16,19). The D1 survey was administered to 2,582 sixt- through eighth-grade students (response rate, 89.8%) during the 2008–2009 school year. The 4 schools were selected because of their different approaches to providing a sports program. Two schools offered only competitive interscholastic sports, and 2 schools offered intramural sports exclusively in their co-curricular programs. The primary measures of sports participation were students’ self-reports of participation in co-curricular sports at school (school sport) or participation in a sport outside of school (outside sport). For the purposes of this analysis, a community sport was an organized sport within a student’s community (ie, in a recreation program, at a YMCA, at a church, or in a youth sports’ organization team).

Although the D1 data represent the most comprehensive individual-level physical activity data collected from middle school students in North Carolina, they include schools offering only 1 type of school sports program. Some schools in the state offer a combination of intramural and interscholastic sports. The first wave of the National Education Longitudinal Survey (NELS), which includes a national sample of schools with different types of sport programs, was used to calculate differences in sports participation rates when intramurals were offered along with interscholastic sports within the same school. This first NELS was conducted in 1988 by the National Center for Education Statistics and includes data obtained from 27,394 eighth-graders in a random national sample of 1,000 middle schools (20).

To simulate physical activity rates based on sports participation, data were obtained from 1,188 observations conducted from April 2009 through December 2009 at the same schools from D1, by using the System for Observing Play and Leisure in Youth (SOPLAY) (21). Trained observers recorded use, location characteristics, and student physical activity levels in the predetermined physical activity areas that were designated for sports in each of the 4 schools, between 2:30 PM and 4:30 PM. SOPLAY uses momentary time sampling to provide a count of individuals within each designated activity zone and classification of observed activity. Standard Metabolic Equivalents (METs) were assigned to SOPLAY codes as 1.5 = sedentary, 3.0 = walking/moderate, and 6.0 = vigorous. An estimate of kilocalories (kcal) per hour was calculated on the basis of Ridley et al (22) by multiplying the average energy expenditure per sport times 43.1 kg (the mean weight for children aged 11, 12, and 13 years, assuming a 50–50 sex ratio per school).

School-level data were integrated from 2 sources. Information related to school-based sport and physical activity programs was obtained from a self-administered Web-based questionnaire completed by personnel at 325 North Carolina middle schools (response rate, 74.4%) in January 2009 (data set 2, [D2]) (7). Respondents received a $20 incentive for completing the survey. The questionnaire was adapted from the School Health Policies and Programs Study 2006 to measure school-based physical activity environments. School population data (ie, enrollment, percentage female, percentage minority, percentage economically disadvantaged) were obtained from publicly available data at the North Carolina Department of Public Instruction (NCDPI).

### Baseline estimates and data analysis

Data were analyzed using SPSS 21.0 (IBM Corp, Armonk, New York). Baseline estimates of state participation rates were established on the basis of data from D1. It was assumed that the participation patterns of sixth- through eighth-graders in this sample would be representative of statewide participation rates. We also assumed equilibrium of supply and demand. That is, this sample represented ideal levels of motivation for sports participation (demand) as well as ideal levels of opportunities (supply) both in school and out of school (eg, public recreation departments). On the basis of analysis of school-level data in D2, the sampled schools offered similar numbers and types of activities within their co-curricular programs as school programs statewide.

For participation rate calculations, a stratified approach was used to estimate sports participation (both school- and community-based sport) on the basis of grade level (sixth grade/seventh and eighth grade), race/ethnicity (white/race other than white), economic status (economically disadvantaged/not economically disadvantaged), and sports program type (intramural sports/interscholastic sports). Grade levels were split in this way because a North Carolina policy prevents sixth-graders from participating in interscholastic sports, thus restricting their access to sports participation. The actual rates of sports participation for each population group in D1 were then used to extrapolate participation rates statewide by applying estimated participation rates to the sample of schools in D2. These rates were then extrapolated to the total student population at all North Carolina middle schools. School-level sports participation rates were estimated for each school in D2 on the basis of its enrollment in each of the stratified groups and sports program type.

To estimate physical activity intensity, standard METs were assigned to each available activity within either an intramural or interscholastic program. Because no direct observations of community sports activities were available, the average of observations of participation within this activity was used (ie, the average METs of intramural soccer and interscholastic soccer = community soccer). By using our estimated statewide sports participation rates, we then calculated rates of participation within each activity among sports participants. We were able to estimate participation rates by activity (ie, type of sport), program delivery type (ie, school intramural, school interscholastic, and community), and style of participation (ie, community only; intramural only; interscholastic only; intramural and interscholastic; intramural and community; interscholastic and community; and intramural, interscholastic, and community). Because participation style and activity type differed significantly by economic status, calculations were stratified by economic status. The number of estimated participants within each activity was then multiplied by the standard MET rate for the activity and then converted to kcal per hour. For nonparticipants, METs were held constant at 1.5 (64.65 kcals/h).

To simulate potential changes in statewide rates of adolescent sports participation, a multiple regression model was estimated by using the simulated school-level sample, controlling for percentage of sixth graders, percentage of minority students, and percentage of economically disadvantaged students. Parameter estimates to calculate estimated variation were generated from existing school program conditions. We recalculated both participation rates and related energy expenditure for schools based on 2 hypothetical scenarios: 1) All schools in the D2 sample adopted policies to offer only intramural sports, and 2) All schools in the D2 sample adopted policies to offer both intramurals and interscholastic sports. Because of the expected influence of economic status on sports participation, the potential effects of policy changes were analyzed separately for students who were economically disadvantaged and those who were not.

## Results

### Baseline estimates

Fifty-three percent of sampled schools (n = 168) offered no intramurals or noncompetitive activities, and of the 47% of schools (n = 149) that do offer these activities, 6.7% (n = 10) do not allow sixth grade participation. Because of state and school policies restricting sixth grade participation, 32,000 sixth- graders in the sample attended schools that provided no access to co-curricular sports.

On the basis of the imputation of stratified estimated sports participation rates, 59.39% of sixth-graders and 63.4% of seventh and eighth graders were estimated to participate in organized sports either at school or in the community. Overall, an estimated 62.3% of middle school students statewide participated in organized sports. Our estimated figure was similar to estimates based on the aggregate results of the North Carolina Youth Risk Behavior Survey that estimated 61.5% of sixth- through eighth- graders participated in sports at or outside school in 2009 (23). On the basis of NCDPI’s report of 330,062 students attending public middle schools, this estimate suggests that approximately 39% (124,502 students) in North Carolina middle schools may not be participating in sports. The current participation rate for students classified as economically disadvantaged was estimated at 52.3% compared with 70.2% for students not classified as economically disadvantaged. Among all sports participants, 37.2% were estimated to be economically disadvantaged.

In terms of physical activity expenditure, we estimated the energy expenditure of sports participants averaged 117.34 kcal per hour during the after-school period, compared with the assumed average of 64.65 kcal per hour for nonparticipants. We estimated the overall energy expenditure per student at an average of 97.56 kcal per hour and an average of 59,481.02 kcal per hour expended daily per school. Economically disadvantaged students averaged 93.40 kcal per hour compared with 100.86 kcal per hour for students not economically disadvantaged.

The baseline regression model used to estimate the effects of changes to participation rates based on policy changes is presented in Table 1. Controlling for the other variables, offering intramural sports had the strongest positive association with higher sports participation rates (increasing rates from 15% to 17%). The percentage of economically disadvantaged students had the strongest negative association with sports participation rates.

**Table 1:** Regression Analysis Predicting Overall Sports Participation Rate by a Sample of Middle School Students (N = 329), North Carolina, 2008–2009

School Characteristic	β Unstandardized Coefficients (SE) (95% CI)	β Standardized Coefficients^a^	T Value	*P* Value
Constant	.608 (.016) (.575 to 640)	NA	36.92	<.001
Sixth-graders (%)	−.029 (.016) (−.059 to −.002)	−.027	−1.82	.069
Race other than white (%)	−.064 (.007) (−.077 to −.050)	−.163	−9.28	<.001
Economically disadvantaged (%)	−.184 (.010) (−.204 to −.165)	−.324	−18.25	<.001
Offers interscholastic sports	.050 (.015) (.020 to 079)	.049	3.29	.001
Offers intramural sports	.161 (.003) (.155 to 167)	.837	56.56	<.001

Abbreviations: SE, standard error; CI, confidence interval; NA, not applicable.

a *R^2^* = .93.

### Simulated effects of policy changes

If all middle schools offered only intramural sports, we estimated there would be a 1.1% increase in existing rates of sports participation. It was further estimated that economically disadvantaged students may benefit more from this policy change than might students who are not economically disadvantaged (Table 2). Policy adoption would increase participation among economically disadvantaged students but decrease participation among students who are not economically disadvantaged. In terms of kcals, this policy implementation was estimated to increase physical activity 4.9% for economically disadvantaged students but only 1.2% for those not economically disadvantaged.

**Table 2:** Simulated Effects if State Adopted a Policy Mandating Only Intramural Sports Programs in All Middle Schools

Student Characteristic	Values Prepolicy and Postpolicy Change	Participation Rate, %	Estimated No. of North Carolina Participants	Kcal Expended Hourly per Student	Kcal Expended Daily per School
Economically disadvantaged	Prepolicy change	52.3	76,386	93.40	25,198.88
	Postpolicy change	56.1	82,009	97.97	26,431.18
	Difference	3.8	5,623	4.57	1,241.3
	Percentage change	7.4	7.4	4.9	4.9

Not economically disadvantaged	Prepolicy change	70.2	129,174	100.86	34,282.14
	Postpolicy change	68.3	125,702	102.06	34,688.43
	Difference	−1.9	−3,472	1.2	406.29
	Percentage change	− 2.7	− 2.7	+1.2	1.2

Total	Prepolicy change	62.3	205,560	97.56	59,481.02
	Postpolicy change	62.9	207,711	100.25	61,119.61
	Difference	0.6	2,151	2.69	1,638.59
	Percentage change	1.1	1.1	2.8	2.8

Abbreviation: kcal, kilocalories

If all middle schools offered both intramural and interscholastic sports, we estimated sports participation could increase by 21.2% and by more than 36% for economically disadvantaged students, resulting in 43,504 new sports participants. More than 28,000 new sports participants (64.5%) were estimated to be economically disadvantaged students. Additionally, we estimated that adopting this policy could yield an increase of 7.66% kcal expenditure per hour per student; and it could yield a 12.12% increase in kcal expenditure per hour for economically disadvantaged students (Table 3).

**Table 3:** Simulated Effects if State Adopted a Policy Mandating Both Intramural and Interscholastic Sports Programs in All Middle Schools

Student Characteristic	Values Prepolicy and Postpolicy Change	Participation Rate	Estimated Number North Carolina Participants	Kcal Expended Hourly by Student	Daily Kcal Expended per School
Economically disadvantaged	Prepolicy change	52.3%	76,386	93.40	25,198.88
	Postpolicy change	71.5%	104,455	104.72	28,252.75
	Difference	19.2	28,069	11.32	3,053.87
	Percentage change	36.8	36.8	12.1	12.1

Not economically disadvantaged	Prepolicy change	70.2%	129,174	100.86	34,282.14
	Postpolicy change	78.6%	144,609	105.27	35,783.23
	Difference	8.4	15,435	4.41	1,501.09
	Percentage change	11.9	11.9	4.4	4.4

Total	Prepolicy change	62.3%	205,560	97.56	59,481.02
	Postpolicy change	75.5%	249,064	105.03	64,035.98
	Difference	13.2	43,504	7.47	4,554.96
	Percentage change	21.2	21.2	7.7	7.7

Abbreviation: kcal, kilocalorie.

## Discussion

Simulations suggest that policy changes to implement intramural sports along with interscholastic sports could result in more than 43,000 new sports participants statewide, of which we estimated 64% would be economically disadvantaged. Substitution of interscholastic sports with intramural sports may have modest positive effects on participation and physical activity. However, offering both types of sports seems to have the most potential for increasing the reach and efficacy of school-based programs. Assuming only 1 hour per day for after-school sports for 180 school days, we estimated that adding intramurals to existing interscholastic sports programs at all middle schools in North Carolina could have an annual effect of 819,892.65 kcals expended above current levels. As suggested by other researchers (15,19), intramural sports should be considered as part of a comprehensive school physical activity program rather than as a substitute for existing sports programs.

Current school policies favoring interscholastic sports may be creating inequitable access to sports and physical activity. Currently, the average economically disadvantaged middle school student in North Carolina is estimated to expend 93.4 kcal per hour, whereas the average for students not economically disadvantaged is 100.86 kcal. Including both interscholastic and intramural sports programs, we estimated those rates would increase to 104.72 kcal and 105.27 kcal, respectively. Providing intramural sports may not only increase overall sports participation and physical activity levels, it also may reduce economic disparities in rates of physical activity by improving access. Additionally, nonparticipants’ METs were held constant at 1.5. It can be speculated that this assumption may have overestimated energy expenditure for nonparticipating economically disadvantaged students who were more sedentary. Thus, differences in physical activity levels uncovered in the simulations may, in reality, be more substantial. Disparities in current rates of sports participation and levels of physical activity were not surprising, considering the well-documented disparities in physical activity among adolescents (16). School environments often offer low-income adolescents the only option for organized sports and physical activity programs (24). However, interscholastic sports, with higher time commitments, skill requirements, and economic costs than intramural sports, may inhibit many interested adolescents from participating (25). The addition of more casual and low-cost opportunities for sports participation at school seems to be a potential mechanism for increasing physical activity, particularly for low-income adolescents.

This simulation approach has inherent limitations. First, we assumed that rates of participation would be uniform across schools and based on the population variables chosen in the baseline schools. Thus, fluctuations related to local factors may not have been captured. However, population variables chosen to model simulated participation rates have been shown, both in previous research and in our analyses, to be theoretically meaningful predictors of sports participation. Additionally, baseline estimates closely matched participation rates collected from a larger sample of students across the state.

Our analysis was also limited to 1 state, and the generalizability of our results may be limited to North Carolina. However, since school policy in the United States is implemented at the state level, this approach may be the most valid to inform policy that affects school environments. The county in which the D1 schools are located ranked near the top of the state in provision of public recreation programs and facilities (26). This suggested that students statewide who were similarly motivated to participate in community sports, particularly students in disadvantaged rural areas of the state, may have fewer opportunities to do so. No reliable measure of effect size was found to estimate the potential variability in participation that could be accounted for by recreation supply outside of school. Therefore, the level of community recreation supply was assumed to be constant across the state. Because of this assumption, baseline estimates of current levels of outside sports participation may be higher than actually exist. Finally, the increased availability of linkable individual-, school-, and community-level factors associated with participation in sports and other physical activity would enhance the validity of our models. Despite the limitations inherent in simulations, their use is encouraged when considering the potential effects of policy decision to improve estimates of policy impact (27).

As resources for school-based sports and other physical activity continue to diminish, school administrators must determine whether potential policy changes will have the desired impact. Our findings suggest the addition of intramural sports as a complement to existing interscholastic sports programs is likely to encourage participation and higher levels of physical activity among a larger segment of the student population. The estimated effect for students from economically disadvantaged backgrounds further suggests that policies promoting intramural sports programs may reduce current economic disparities in physical activity. Because implementation of intramural sports is less expensive and labor intensive than other interventions, the benefits of this policy change warrant further investigation.

## References

[1] Ogden CL , Carroll MD , Kit BK , Flegal KM . Prevalence of obesity and trends in body mass index among US children and adolescents, 1999-2010. JAMA 2012;307(5):483–90. 10.1001/jama.2012.40 22253364 PMC6362452

[2] World Health Organization. Health and development through physical activity and sport. Geneva (CH): WHO Document Production Services; 2003. http://whqlibdoc.who.int/hq/2003/WHO_NMH_NPH_PAH_03.2.pdf . Accessed February 16, 2013.

[3] Paluska SA , Schwenk TL . Physical activity and mental health. Sports Med 2000;29(3):167–80. 10.2165/00007256-200029030-00003 10739267

[4] Sirard JR , Pfeiffer KA , Pate RR . Motivational factors associated with sports program participation in middle school students. J Adolesc Health 2006;38(6):696–703. 10.1016/j.jadohealth.2005.07.013 16730598

[5] Nelson TF , Stovitz SD , Thomas M , LaVoi NM , Bauer KW , Neumark-Sztainer D . Do youth sports prevent pediatric obesity? A systematic review and commentary. Curr Sports Med Rep 2011;10(6):360–70. 10.1249/JSR.0b013e318237bf74 22071397 PMC4444042

[6] Perkins DF , Jacobs JE , Barber BL , Eccles JS . Childhood and adolescent sports participation as predictors of participation in sports and physical fitness activities during young adulthood. Youth Soc 2004;35(4):495–520. 10.1177/0044118X03261619

[7] Edwards MB , Bocarro JN , Kanters MA . Place disparities in access to supportive environments for extracurricular physical activity in North Carolina middle schools. Youth Soc 2013;45(2):265–85. 10.1177/0044118X11416677

[8] Johnston LD , Delva J , O’Malley PM . Sports participation and physical education in American secondary schools: current levels and racial/ethnic and socioeconomic disparities. Am J Prev Med 2007;33(4 Suppl):S195–208. 10.1016/j.amepre.2007.07.015 17884568

[9] Casper JM , Bocarro JN , Kanters MA , Floyd MF . “Just let me play!” Understanding constraints that limit adolescent sport participation. J Phys Act Health 2011;8 Suppl 1:S32–9. 21350260 10.1123/jpah.8.s1.s32

[10] Lee SM , Burgeson CR , Fulton JE , Spain CG . Physical education and physical activity: results from the School Health Policies and Programs Study 2006. J Sch Health 2007;77(8):435–63. 10.1111/j.1746-1561.2007.00229.x 17908102

[11] Holt NL , Kingsley BC , Tink LN , Scherer J . Benefits and challenges associated with sport participation by children and parents from low-income families. Psychol Sport Exerc 2011;12(5):490–9. 10.1016/j.psychsport.2011.05.007

[12] Reeves K . Sports at any cost? Sch Administrator 2006;63(6):28–34.

[13] Leek D , Carlson JA , Cain KL , Henrichon S , Rosenberg D , Patrick K , Physical activity during youth sports practices. Arch Pediatr Adolesc Med 2011;165(4):294–9. 10.1001/archpediatrics.2010.252 21135319

[14] Wickel EE , Eisenmann JC . Contribution of youth sport to total daily physical activity among 6- to 12-yr-old boys. Med Sci Sports Exerc 2007;39(9):1493–500. 10.1249/mss.0b013e318093f56a 17805079

[15] Koplan JP , Liverman CT , Kraak VI . Preventing childhood obesity: health in the balance. Washington (DC): The National Academies Press; 2005. 22379642

[16] Kanters MA , Bocarro JN , Edwards MB , Casper JM , Floyd MF . School sport participation under two school sport policies: comparisons by race/ethnicity, gender, and socioeconomic status. Ann Behav Med 2013;45 Suppl 1:S113–21. 10.1007/s12160-012-9413-2 22993023

[17] National Association for Sport and Physical Education. Before- and after-school physical activity and intramural sport programs. Reston (VA): National Association for Sport and Physical Education, an Association of the American Alliance for Health, Physical Education, Recreation and Dance; 2013. http://www.aahperd.org/naspe/standards/positionStatements/upload/Guidelines-Before-After-School-PA-Intramurals-Draft-for-Board-11-27-12-2.pdf. Accessed September 5, 2013.

[18] Campbell M , Fitzpatrick R , Haines A , Kinmonth AL , Sandercock P , Spiegelhalter D , Framework for design and evaluation of complex interventions to improve health. BMJ 2000;321(7262):694–6. 10.1136/bmj.321.7262.694 10987780 PMC1118564

[19] Bocarro JN , Kanters MA , Cerin E , Floyd MF , Casper JM , Suau LJ , School sport policy and school-based physical activity environments and their association with observed physical activity in middle school children. Health Place 2012;18(1):31–8. 10.1016/j.healthplace.2011.08.007 21900034

[20] National Center for Education Statistics. National Education Longitudinal Study of 1988 (NELS: 88). http://nces.ed.gov/surveys/nels88/ . Accessed July 4, 2011.

[21] McKenzie TL . The use of direct observation to assess physical activity. In: Welk G, editor. Physical activity assessments for health-related research. Champaign (IL): Human Kinetics; 2002. p. 179-96.

[22] Ridley K , Ainsworth B , Olds T . Development of a compendium of energy expenditures for youth. Int J Behav Nutr Phys Act 2008;5:45. 10.1186/1479-5868-5-45 18782458 PMC2564974

[23] Healthy Schools NC. N.C. Youth Risk Behavior Survey (YRBS). Department of Public Instruction and Department of Health and Human Services. www.nchealthyschools.org/data/yrbs. Accessed August 29, 2011.

[24] Outley CW , Floyd MF . The home they live in: inner city children’s views on the influence of parenting strategies on their leisure behavior. Leis Sci 2002;24(2):161–79. 10.1080/01490400252900130

[25] Edwards MB , Kanters MA , Bocarro JN . Opportunities for extracurricular physical activity in North Carolina middle schools. J Phys Act Health 2011;8(5):597–605. 21734304 10.1123/jpah.8.5.597

[26] Edwards MB , Jilcott SB , Floyd MF , Moore JB . County-level disparities in access to recreational resources and associations with adult obesity. J Park Recreat Admin 2011;29(2):39–54.

[27] Homer J , Milstein B , Wile K , Pratibhu P , Farris R , Orenstein DR . Modeling the local dynamics of cardiovascular health: risk factors, context, and capacity. Prev Chronic Dis 2008;5(2):A63. 18341798 PMC2396963

